# First Complete Genome Sequence of *Potato leafroll virus* from Argentina

**DOI:** 10.1128/genomeA.00628-17

**Published:** 2017-07-27

**Authors:** María P. Barrios Barón, Yamila C. Agrofoglio, Verónica C. Delfosse, Vanesa Nahirñak, Martín Gonzalez de Urreta, Natalia I. Almasia, Cecilia Vazquez Rovere, Ana J. Distéfano

**Affiliations:** a Instituto de Biotecnología, INTA-CICVyA, CONICET, Buenos Aires, Argentina; b Universidad Nacional de San Martin, Buenos Aires, Argentina

## Abstract

In this study, we determined for the first time the complete genomic sequence of an Argentinian isolate of *Potato leafroll virus* (PLRV), the type species of the genus *Polerovirus*. The isolate sequenced came from a *Solanum tuberosum* plant that had been naturally infected with the virus. Isolate PLRV-AR had a nucleotide sequence identity between 94.4 and 97.3% with several known PLRV isolates worldwide.

## GENOME ANNOUNCEMENT

*Potato leafroll virus* (PLRV) is the type species of the genus *Polerovirus* within the family *Luteoviridae*, which is a group of phloem-limited plant viruses transmitted efficiently by a limited number of aphid species (1). Particularly, the green peach aphid, *Myzus persicae*, is the most efficient and important vector of PLRV (1, 2). PLRV has a single-strand positive-sense RNA genome of about 5.9 kb with 10 open reading frames (ORFs) (1). PLRV sequences worldwide are very closely related and show low genetic diversity (3). PLRV is considered one of the most damaging potato (*Solanum tuberosum*) viruses and causes significant yield and quality losses to infected potato plants worldwide (4). This virus produces an important viral disease in Argentina, and it is distributed within all major potato-growing regions.

So far, the ORF2 sequence of a PLRV isolate, which was detected in 1999, is the only partial sequence reported of an Argentinian PLRV isolate (5). To obtain a complete genome sequence of an Argentinian isolate of PLRV, we obtained a field-grown infected *S. tuberosum* cv. Kennebec plant from Tupungato, Mendoza, in 2013. Total RNA was isolated from infected leaf samples using the TriPure isolation reagent (Roche Molecular Systems, Inc., USA), and cDNA was then synthesized with reverse transcriptase SuperScript III (Thermo Fisher Scientific, Inc., USA). This cDNA was used as a template for the subsequent PCRs, whose primer pairs were designed based on the Dutch PLRV genome sequence (GenBank accession no. Y07496). Five overlapping PCR fragments, which covered almost the complete viral genome, were amplified by using FideliTaq (Thermo Fisher Scientific, Inc.); 5′ and 3′ ends were amplified by 5′ and 3′ rapid amplification of cDNA ends (RACE) (Thermo Fisher Scientific, Inc.). The amplicons obtained were gel purified (QUIAEX II [Qiagen, Germany]), cloned into a pGEM-T easy vector (Promega, USA), and sequenced by the Sanger methods in a Genetic Analyzer 3500XL (Applied Biosystems) with at least 2-fold coverage in both orientations. Sequencing data were assembled and analyzed with the Vector NTI software (Thermo Fisher Scientific, Inc.).

The genome of PLRV-AR contains 5,881 nucleotides, and its deduced genome organization resembled other PLRV isolates. The genome has 10 ORFs coding for proteins P0, P1, Rap1, P1-P2, P3a, P3, P4, P3-P5, P6, and P7. The entire nucleotide sequence was compared to other PLRV sequences reported and available in GenBank (http://www.ncbi.nlm.nih.gov/Genbank/index.html). The identity of PLRV-AR with the compared isolates ranged between 94.4 and 97.3%. This finding confirms low variability of this virus, as previously reported; in addition, this variability is not correlated with the geographical origin of the isolate. PLRV-AR showed maximum identity (97.3%) with a Polish isolate (GenBank accession no. X74789) and minimum identity (94.4%) with an isolate from Australia (GenBank accession no. D13953), which was previously reported as the most divergent among the PLRV isolates.

To our knowledge, this is the first report of a complete genome sequence of an Argentinian PLRV isolate.

### Accession number(s).

The complete genome sequence of PLRV-AR has been deposited in GenBank under the accession number KY856831.
